# Tolfenamic Acid Induces Apoptosis and Growth Inhibition in Head and Neck Cancer: Involvement of NAG-1 Expression

**DOI:** 10.1371/journal.pone.0034988

**Published:** 2012-04-19

**Authors:** Sung Un Kang, Yoo Seob Shin, Hye Sook Hwang, Seung Joon Baek, Seong-Ho Lee, Chul-Ho Kim

**Affiliations:** 1 Department of Otolaryngology, School of Medicine, Ajou University, Suwon, Korea; 2 Center for Cell Death Regulating Biodrug, School of Medicine, Ajou University, Suwon, Korea; 3 Department of Biomedical and Diagnostic Sciences, College of Veterinary Medicine, University of Tennessee, Knoxville, Tennessee, United States of America; 4 Department of Nutrition and Food Science, College of Agriculture and Natural Resources, University of Maryland, College Park, Maryland, United States of America; Henry Ford Health System, United States of America

## Abstract

Nonsteroidal anti-inflammatory drug-activated gene-1 (NAG-1) is induced by nonsteroidal anti-inflammatory drugs and possesses proapoptotic and antitumorigenic activities. Although tolfenamic acid (TA) induces apoptosis in head and neck cancer cells, the relationship between NAG-1 and TA has not been determined. This study investigated the induction of apoptosis in head and neck cancer cells treated by TA and the role of NAG-1 expression in this induction. TA reduced head and neck cancer cell viability in a dose-dependent manner and induced apoptosis. The induced apoptosis was coincident with the expression of NAG-1. Overexpression of NAG-1 enhanced the apoptotic effect of TA, whereas suppression of NAG-1 expression by small interfering RNA attenuated TA-induced apoptosis. TA significantly inhibited tumor formation as assessed by xenograft models, and this result accompanied the induction of apoptotic cells and NAG-1 expression in tumor tissue samples. Taken together, these results demonstrate that TA induces apoptosis via NAG-1 expression in head and neck squamous cell carcinoma, providing an additional mechanistic explanation for the apoptotic activity of TA.

## Introduction

With over 500,000 cases worldwide and a high mortality rate, head and neck squamous cell carcinoma (HNSCC) is the sixth most common cancer in men. Even with improved treatments, the overall survival rate has been under 50% for the past 30 years [1]. Although some patients achieve long-term survival, particularly those diagnosed with early-stage disease, most patients with this type of cancer have advanced disease at the time of diagnosis. These patients run a risk of recurrent disease, distant metastases, or second primary tumors, and have a median survival of only 6–8 months [2]

The 5-year overall survival rate for patients with advanced HNSCC is around 30%, which is essentially unchanged from the rate recorded two decades ago in spite of various treatment trials. Therefore, preventive strategies are desirable, and much research is currently devoted to chemoprevention that aims to prevent HNSCC progression at an early stage. However, which chemopreventive agents are the safest and most effective against HNSCC remains unclear. It is of clinical importance to investigate the efficacy of nonsteroidal anti-inflammatory drugs (NSAIDs) as chemopreventive agents.

Chemopreventive agents play an important role in interrupting the carcinogenic process and inhibiting the recurrence of precancerous lesions. At present, the pharmaceutical agents that have been most widely investigated as chemopreventive agents are NSAIDs. NSAIDs have been clinically used as chemopreventive agents for familial adenomatous polyposis, with the mode of action being the inhibition of cyclooxygenase-2 (COX-2) [3], [4]. However, chemopreventive and antitumorigenic activities of NSAIDs have also been observed in COX-2-deficient cells, indicating that NSAIDs also exert their anticancer effect through a mechanism other than COX-2 suppression. One such mechanism involves the induction of NSAID-activated gene-1 (NAG-1) [5].

NAG-1 was identified from indomethacin (a COX inhibitor)-induced gene library [5]. NAG-1 is a member of the transforming growth factor-beta (TGF-β) superfamily and is also known as macrophage inhibitory cytokine-1 (MIC-1), growth differentiation factor 15 (GDF15), and prostate derived factor (PDF) [6], [7], [8]. Purified recombinant NAG-1 is able to inhibit lipopolysaccharide-induced tumor necrosis factor-alpha (TNF-α) production in macrophages, suggesting that NAG-1 acts as an autocrine regulatory molecule [9]. *In vitro* and *in vivo*, NAG-1 has anti-tumorigenic and pro-apoptotic activities independent of COX inhibition [5], [10]. NSAIDs and several antitumorigenic compounds with chemopreventive activities, including resveratrol, genistein, catechins, and peroxisome proliferator-activated receptor-*γ* ligands, regulate NAG-1 expression in a prostaglandin-independent manner [11], [12], [13], [14], [15].

However, until now, the relationship, if any, between NAG-1 and TA in HNSCC has not been investigated. Pro-apoptotic activity of NAG-1 may provide a molecular basis to explain chemopreventive agent, TA-mediated antitumorigenesis.

In this study, we examined the regulation of NAG-1 expression during TA-induced apoptosis in HNSCC cells. NAG-1 small interfering RNA (siRNA)-mediated inhibition of NAG-1 expression attenuated TA-induced apoptosis. Furthermore, the *in vivo* antitumorigenic activity of TA in mice was investigated to evaluate the use of TA as a chemopreventive agent in HNSCC. The data suggest that the induction of NAG-1 may provide a novel mechanism for understanding the downstream effectors for TA-induced apoptosis in HNSCC.

## Materials and Methods

### Cell lines and reagents

Four established human head and neck cancer cell lines – KB (an oral cancer cell line), SCCQLL1 (an oral cancer cell line), HN3 (a laryngeal cancer cell line), and FaDu (a hypopharyngeal cancer cell line) – were obtained from American Type Culture Collection (Manassas, VA) and Korean Cell Line Bank (Seoul, Korea). The cells were grown in Dulbecco's modified Eagle's medium with 10% fetal bovine serum and penicillin-streptomycin at 100 U/ml (GIBCO, Carlsbad, CA) at 37°C in a humidified atmosphere with 5% CO_2_ and 95% air. TA, diclofenac, sulindac sulfide, indomethacin, and piroxicam (Sigma-Aldrich, St. Louis, MO) were dissolved in autoclaved water as stock solution for *in vitro* studies. For investigate the effect of TA on normal cells, we used HaCaT cells (normal keratinocytes) that were obtained from Korean Cell Line Bank (Seoul, Korea).

### Cell proliferation assay

Cell viability was tested using an assay based on the conversion of 3-(4,5-dimethylthiazol-2-yl)-2,5-diphenyl-tetrazolium bromide (MTT; Sigma Aldrich). MTT solution was added to 40 µl of cell suspension for 4 h. After three washes with phosphate buffered saline (PBS, pH 7.4), the insoluble formazan product was dissolved in 100 µl of dimethylsulfoxide. Optical density (OD) was measured for each culture well using a microplate reader (Bio-Tek, Winooski, VT) at 540 nm. The OD of control cells were taken as 100% viability.

### Terminal deoxynucleotidyl transferase-mediated dutp-biotin nick end labeling (tunel) assay

DNA fragmentation was analyzed with the *in situ* cell death detection kit (Roche Molecular Biochemicals, Basel, Switzerland), according to the instructions of the manufacturer. The stained cells were analyzed under a fluorescence microscope (Carl Zeiss, Oberkochen, Germany).

### Flow cytometry detection of apoptosis

Apoptosis was detected using the Annexin V-fluorescein isothiocynate (FITC) apoptosis detection kit (BD Biosciences, Bedford, MA). Briefly, cells were plated in 6-well culture dishes and incubated with 0, 10, 20, and 40 µM TA for 24 h. The cells were harvested, washed with PBS, and stained with Annexin V-FITC and propidium iodide (PI). Early and late apoptosis were quantified according to the manufacturer's instructions. Apoptosis was detected using a FACS Canto system (BD Biosciences, Bedford, MA), with excitation and emission settings of 488 and 530 nm, respectively.

### Mitochondrial membrane potential (MMP) assay

MMP of intact cells was measured by flow cytometry with the lipophilic cationic probe 5,5 V,6,6 V-tetrachloro-1,1 V 3,3 V-tetra ethylbenzimidazolcarbocyanine iodide (JC-1; Molecular Probes, Eugene, OR). The culture medium was briefly removed from the adherent KB cells, and the cells were rinsed with PBS. Cell monolayers were incubated with DMEM and 5 µg/ml JC-1 at 33°C for 20 min. The cells were subsequently washed twice with cold PBS and trypsinized. Cell pellets were then resuspended in 500 µl PBS. The change in MMP was measured by flow cytometry (BD Biosciences) and fluorescence microscopy at 72 h after irradiation.

### Transfectiom of NAG-1 cDNA and RNA interference (rnai)

All transfection experiments were performed using Lipofectamine 2000 reagent (Invitrogen, Carlsbad, CA) according to the manufacturer's instructions, as described previously [5]. After incubation for 24 h, the medium was removed and the cells were washed with PBS and treated with either TA or vehicle for 24 h. NAG-1 cDNA was previously described [5]. siRNA for control and NAG-1(sense CUCAGUUGUCCUGCCCUGUdTdT and antisense ACAGGGCAGGACAACUGAGdTdT) were purchased from Invitrogen.

### Western blot analysis

Cells were washed with cold PBS and suspended in RIPA buffer (Sigma-Aldrich) supplemented with PhosSTOP and Complete Mini EDTA-free (Roche Molecular Biochemicals, Basel, Switzerland). The proteins from KB cells were electrotransferred to Immobilon-P membranes (Millipore, Bedford, MA). Detection of specific proteins was carried out with an enhanced chemiluminescence Western blotting kit according to the manufacturer's instructions.

### Animal study

KB cells (1×10^6^) re-suspended in PBS were subcutaneously administered to the lower-right flank of each BALB/c nu/nu mouse. After 1 week, when tumors reached approximately 100 mm in diameter, the mice were randomly divided into two groups (n = 10 per group) and treatment was initiated. Treatment was started the day after cell implantation with either intraperitoneal injection of TA (25 mg/kg) daily (treatment groups) or saline alone (control group). Tumors were measured with a sliding caliper once every 2 days and the volumes (mm^3^) were calculated according to the formula V = A×B^2^×0.52, in which A is the largest superficial diameter and B is the smallest superficial diameter [16]. Animals were euthanized, and tumors were collected and weighed 17 days after implantation. Half of the tissue was snap-frozen in liquid nitrogen and used for protein extraction. The other half was fixed overnight in neutral buffered formalin and processed by routine methods. Western blotting of NAG-1 was performed on tumor specimens and normal soft tissue samples. This study was approved by the Committee for Ethics in Animal Experiments of the Ajou University School of Medicine.

### Statistical analyses

Where data were derived from three independent experiments, the parameters were expressed as mean ± SD. Comparison of the means of different groups was made using one-way analysis of variance. The Student-Newman-Keuls test was used for pair-wise comparisons of results found to be significant by repeated measures of ANOVA. Statistical significance was inferred at *p*<0.05.

## Results

### Induction of NAG-1 and apoptosis in HNSCC cells by various NSAIDs

**Figure 1:**
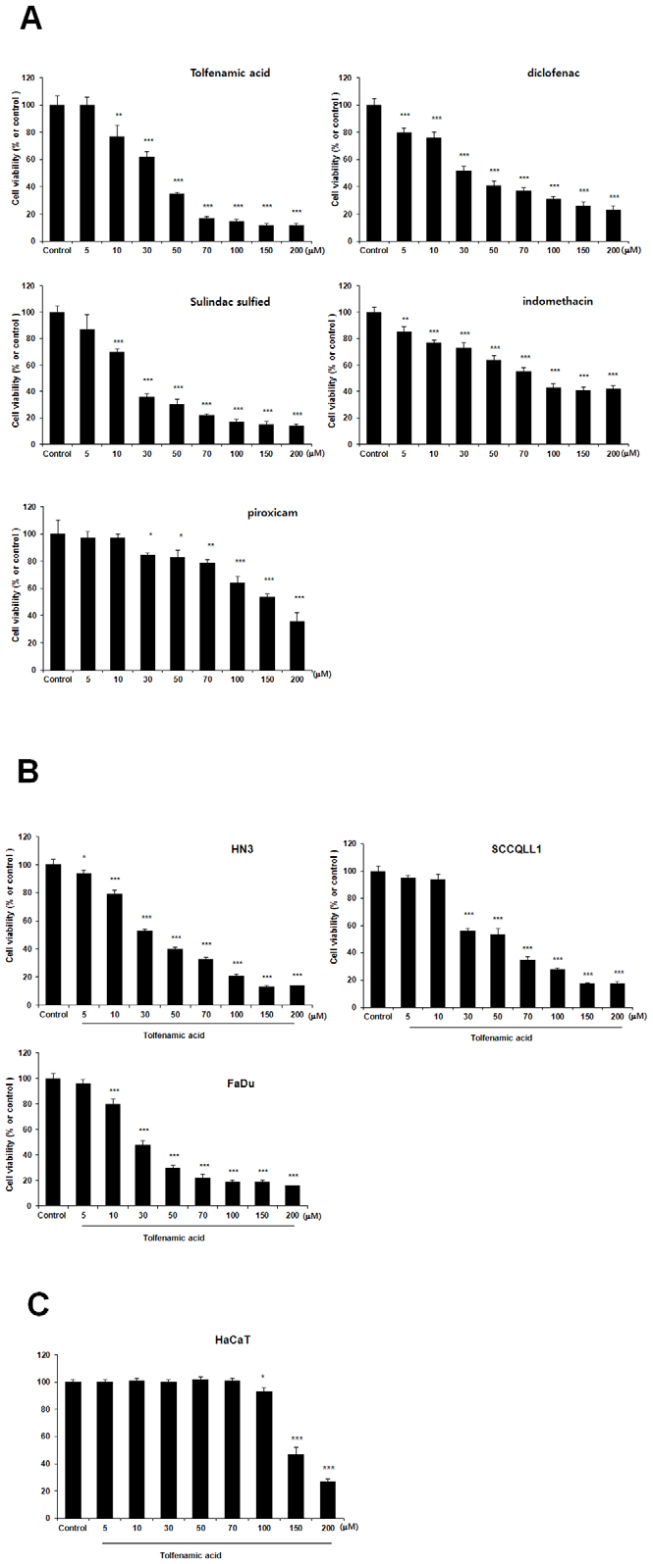
Effect of various NSAIDs on proliferation of various head and neck cancer cell lines and normal keratinocytes. Results of the proliferation assay. Cells were plated in 96-well tissue culture plates at 1000 cells/well in a final volume of 100 µl of medium and were allowed to attach for 2 days. The cells were then treated once with varying doses of TA for 24 h. Cell proliferation was estimated by the MTT assay. Values represent the mean ± S.D. from five independent experiments. * *p*<0.05; ** *p*<0.01, compared with the control group. (A) The KB head and neck cancer cell line was treated with NSAIDs. (B) Cytotoxicity of TA on various HNSCC cells. (C) Cytotoxicity of TA on normal keratinocyte cells (HaCaT).

**Figure 2:**
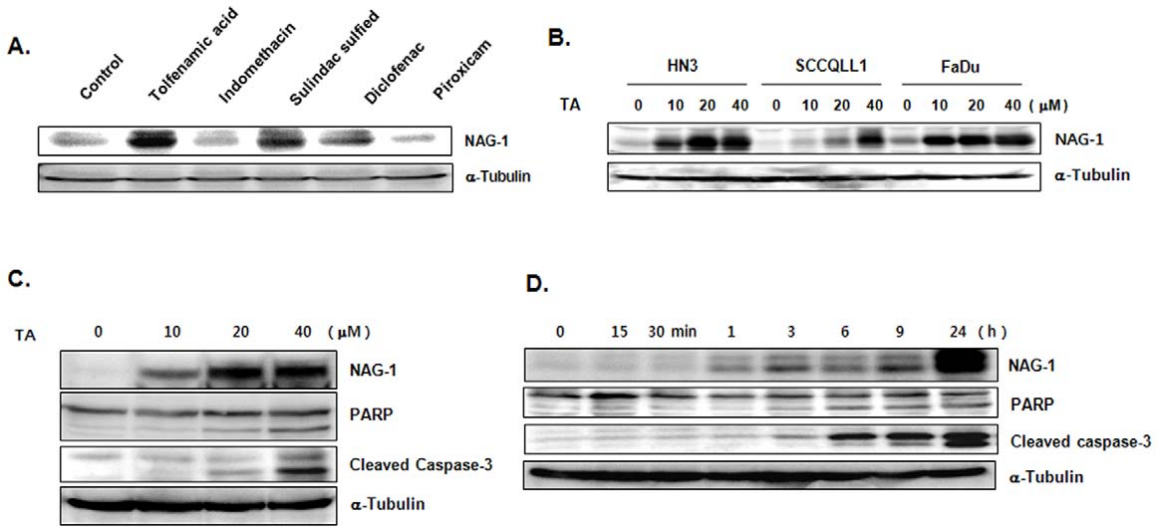
Induction of NAG-1 expression in head and neck cancer cell lines by various NSAIDs. (A) NAG-1 expression was induced by NSAIDs in the following order: TA, indomethacin, sulindac sulfide, diclofenac, and piroxicam. Expression of NAG-1 was measured by Western blot analysis and normalized to the level of a-tubulin expression. (B) Induction of NAG-1 expression in various head and neck cancer cell lines by TA. (C and D) TA dose- and time-dependently increases NAG-1 protein levels in KB cells. KB cells were treated with 0, 10, 30, and 40 µM TA for 24 h. Expression of NAG-1 was measured by Western blot analysis and α–tubulin was used as loading control. The same membrane was stripped and re-probed with cleaved PARP and cleaved caspase-3 antibody.

To investigate the effects of NSAIDs on the growth of HNSCC cells, the four selected HNSCC cell lines were incubated with varying concentrations (0, 5, 10, 30, 50, 70, 100, 150, and 200 µM) of TA for 16 h and cell viability was measured by the MTT assay. As shown in Fig. 1, various NSAIDs reduced cell viability in a dose-dependent manner (Fig. 1A) and TA significantly reduced viability of the HNSCC cell lines (Fig. 1B). Especially, TA was the most potent inducer both apoptosis and NAG-1 expression (Figs. 1 and 2), and was selected for further research. Next, to investigate the toxic effects of TA on normal cells, we performed cell proliferation assay in HaCaT cells (normal keratinocytes). HaCaT cells were treated with TA for 16 h at the indicated concentrations ( 0, 5, 10, 30, 50, 70, 100, 150, 200 µM). The relative viability of the HaCaT cells was examined using the MTT assay (Fig. 1C). TA was minimally cytotoxic on HaCaT upto 100 µM, but TA was significant cytotoxic on the cells at the high concentrations.

**Figure 3:**
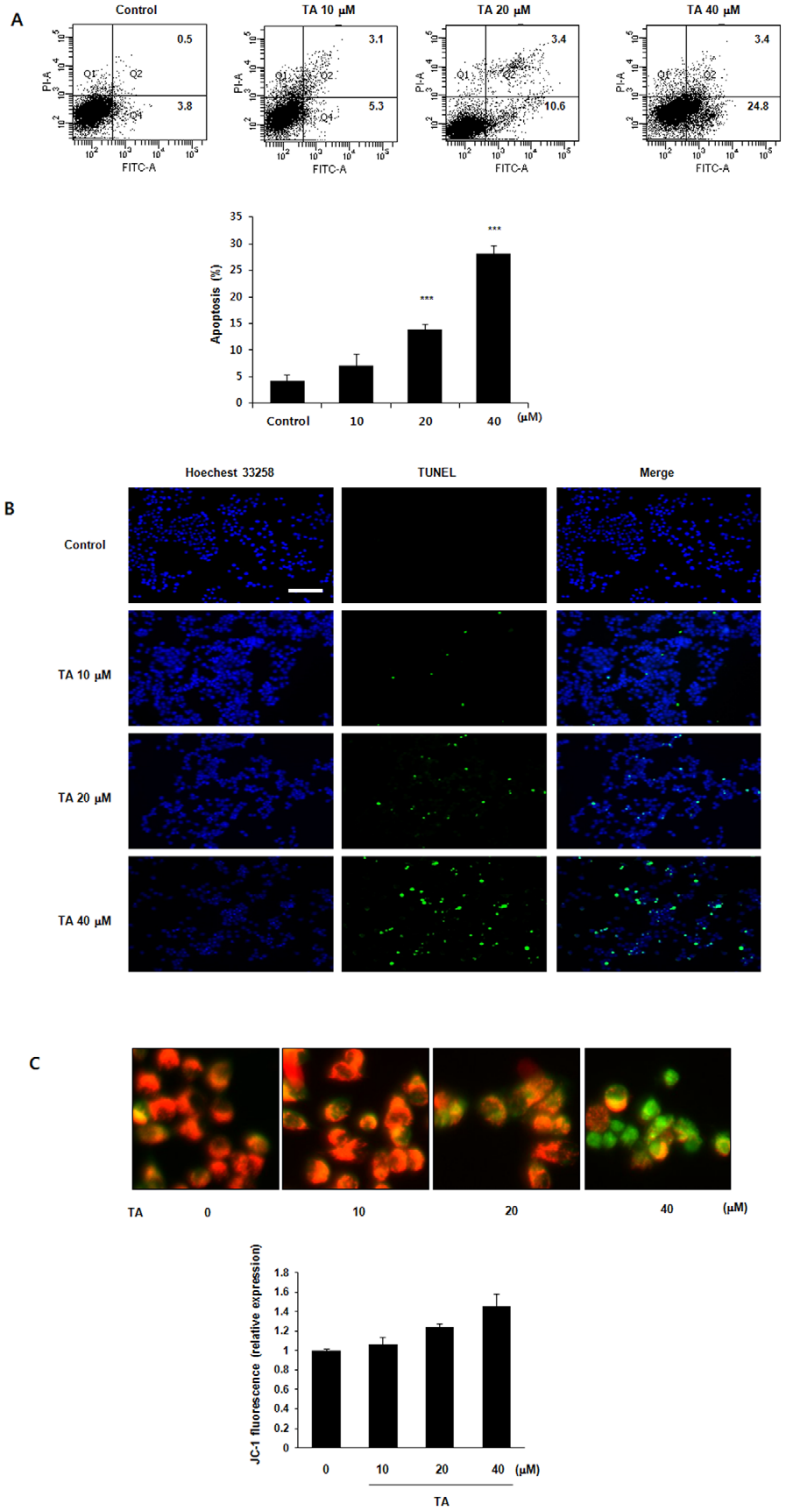
Effect of TA on apoptosis and mitochondrial membrane potential in KB cells. (A) For flow cytometric detection of apoptotic cells, cells were plated in 6-well culture dishes and incubated with 0, 10, 20, 30 and 40 µM TA for 24 h. The cells were harvested and washed with PBS and then stained with Annexin V-FITC and propidium iodide (PI). Early and late apoptosis were quantified. Data are mean values obtained from three independent experiments and bars represent standard deviations. (B) Results of TUNEL analysis. Apoptosis in KB cells was determined by the TUNEL method using an in situ cell detection kit. Cells were treated with indicated concentrations of TA for 24 h. TUNEL staining was increased in apoptotic cell death. Left, 4′,6-diamidino-2-phenylindole (DAPI); middle, TUNEL; right, merge. The scale bar denotes 50 µm. (C) The effect of TA on the mitochondrial apoptotic pathway. After applying 10, 20, 30, and 40 µM TA for 24 h, the JC-1 fluorescence shifted from red-orange to green, indicating depolarization of the mitochondrial membrane potential (MMP). The MMP change was objectively measured using flow cytometry. The data represent the mean ± SD of three independent experiments. *, *P*<0.05; **, *P*<0.01; ***, *P*<0.001 compared with the control group.

To explore the relationship between the NSAID-induced apoptosis and NAG-1 expression, HNSCC cells were treated with various concentrations of NSAID and protein levels of NAG-1 were measured by Western blot analysis. As shown in Fig. 2, various NSAIDs induced the expression of NAG-1 protein in KB cells (Fig. 2A) and TA induced expression of NAG-1 in various HNSCC cells (Fig. 2B). TA also induced expression of NAG-1 protein in dose and time-dependent manners (Figs. 2C and 2D). When cells were treated with 40 µM of TA for various times, induction of NAG-1 protein was seen after 3 h in KB cells (Fig. 2D). The same membrane was stripped and re-probed for cleaved poly (ADP-ribose) polymerase (PARP) and cleaved caspase-3, which were also induced by TA (Figs. 2C and 2D). Overall, these results suggest that TA-induced apoptotic cell death and cell growth arrest in HNSCC cell lines is probably mediated by the expression of the pro-apoptotic protein NAG-1. To determine whether the TA-induced cell death was by apoptosis, we performed fluorescence-activated cell sorting (FACS) with Annexin-V/PI staining and TUNEL assay. As shown in Fig. 3, Annexin V-FITC-positive cells were also increased by TA treatment in KB cells (Fig. 3A) and TUNEL-positive cells by TA increased in a dose-dependent manner (0, 10, 20, and 40 µM) in KB cells (Fig. 3B). MMP can be used as an index of mitochondrial pore opening, which is an indicator of mitochondrial dysfunction. Quantitative analysis of red and green fluorescent signals from intracellular JC-1 dye reflects the degree of mitochondrial damage. Therefore, we examined the effect of TA on MMP in the KB cells to determine whether the loss of MMP could play a role in TA-induced apoptosis. As shown in Fig. 3C, a high MMP was maintained in control cells, as indicated by the predominantly red fluorescence of the JC-1 dye. However, TA treatment increased the green cell fluorescence indicating a loss of MMP and mitochondrial damage (Fig. 3C).

### Overexpression and suppression of NAG-1 promotes and attenuates TA-induced apoptosis, respectively

**Figure 4:**
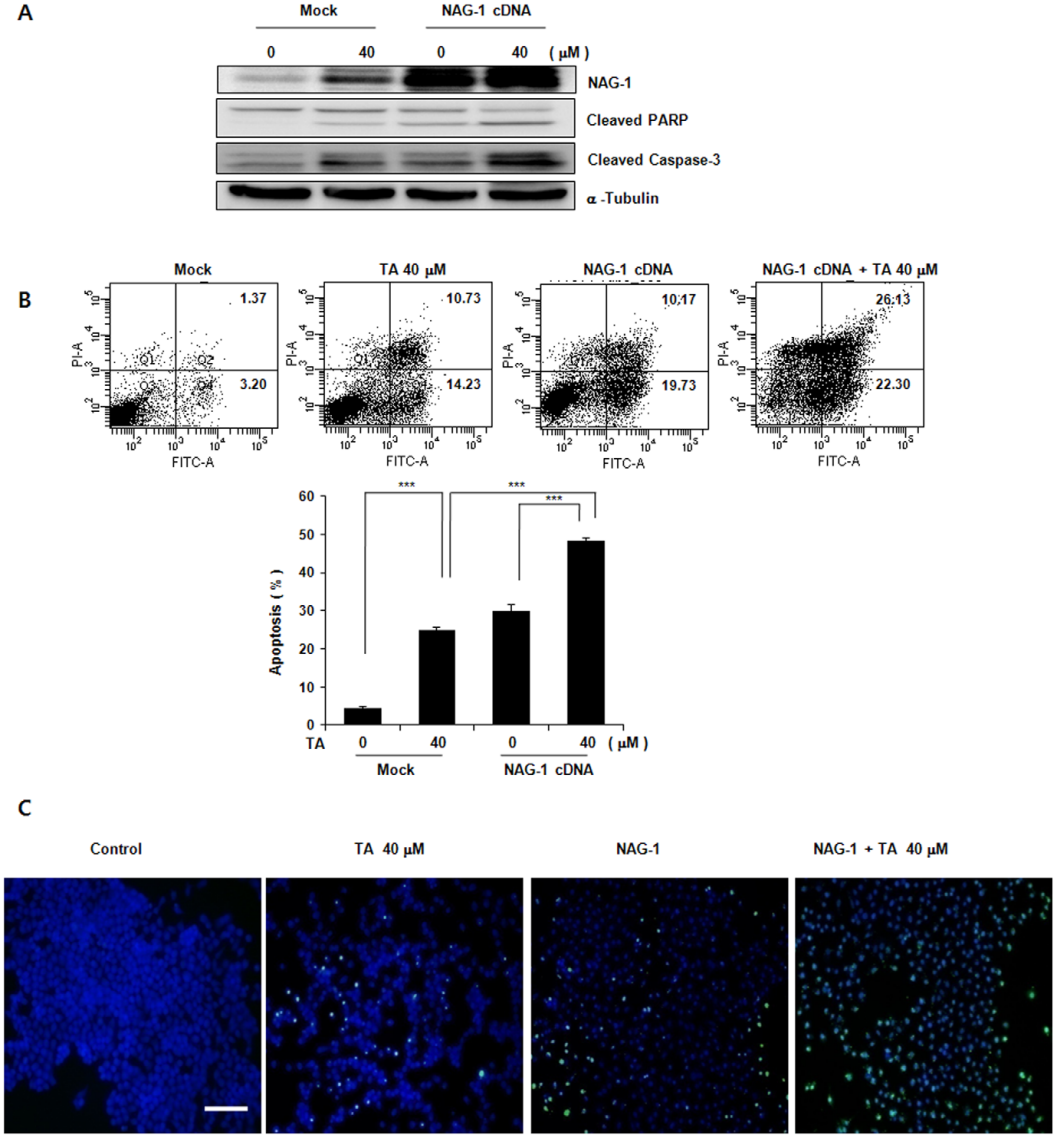
Effect of enhanced expression of NAG-1 on TA-induced apoptosis in KB cells. KB/NAG-1 and KB/vector cells were treated with 30 µM TA for 24 h, and apoptosis was analyzed using a TUNEL assay and by FACS with annexin V-FITC/PI. (A) Western Blot. Equal amounts of cell lysates (30 µg) were resolved by sodium dodecyl sulfate-polyacrylamide gel electrophoresis (SDS-PAGE), transferred to nitrocellulose membrane, and probed with anti-NAG-1, PARP, cleaved caspase-3, or α–tubulin antibody. (B and C) Apoptosis in KB cells was determined by the TUNEL method and FACS with AnnexinV-FITC/PI, as described in Fig. 3. Data are mean values obtained from three independent experiments and bars represent standard deviations. The scale bar denotes 50 µm.

**Figure 5:**
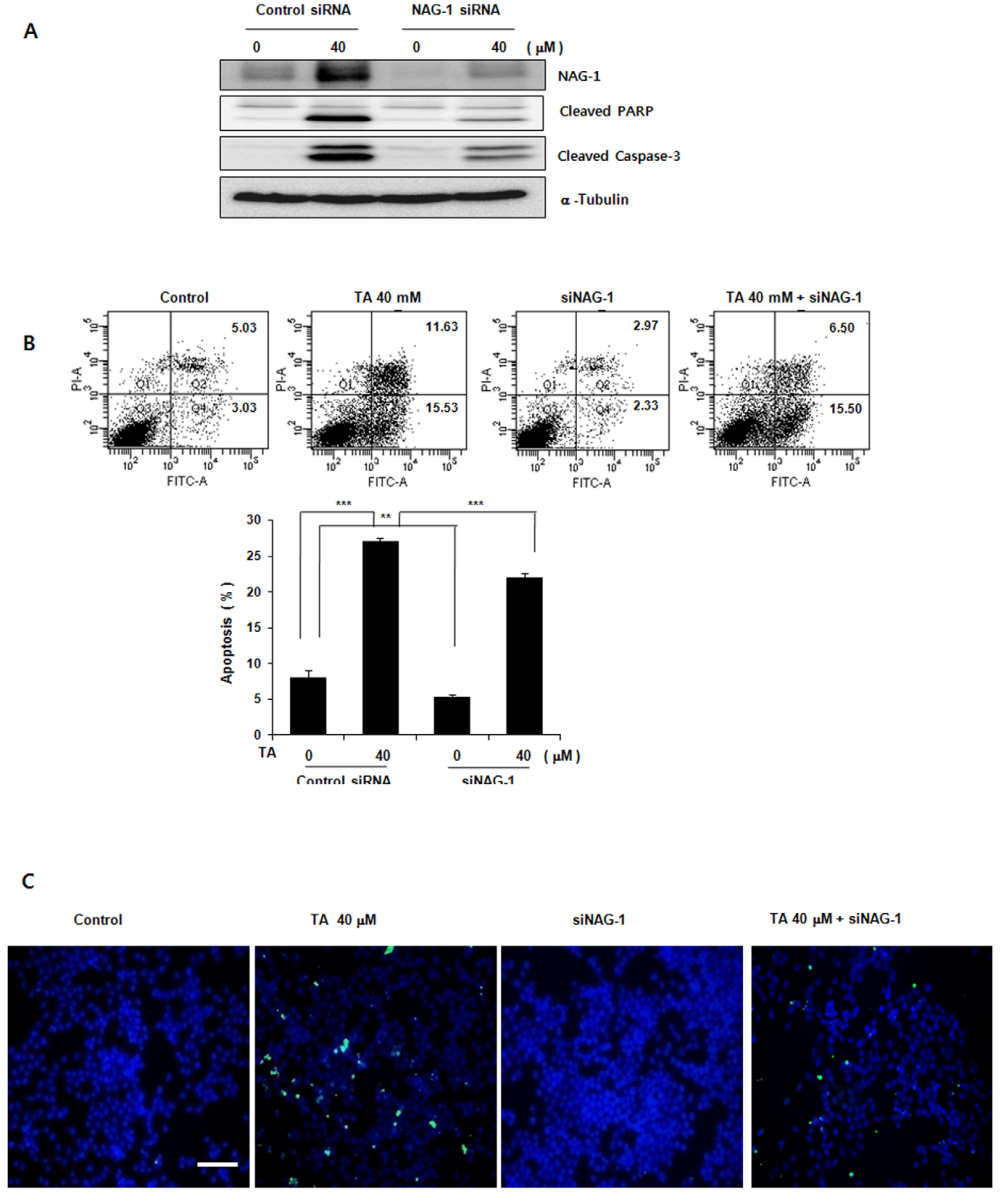
Effect of suppression of NAG-1 expression on TA-induced apoptosis in KB cells. KB cells transfected with either the indicated NAG-1 siRNA or negative control siRNA were treated with or without 30 µM TA for 24 h. Apoptosis was analyzed by the TUNEL assay and by FACS with AnnexinV-FITC/PI. (A) Western Blot. Equal amounts of cell lysates (30 µg) were resolved by SDS-PAGE, transferred to nitrocellulose membrane, and probed with anti- NAG-1, PARP, cleaved caspase-3, orαα–tubulin. (B and C) Apoptosis in KB cells was determined by the TUNEL method and FACS with AnnexinV-FITC/PI, as described in Fig. 3. Data are mean values obtained from three independent experiments and bars represent standard deviations. The scale bar denotes 50 µm.

Next, we attempted to clarify the significance of NAG-1 up-regulation in TA-induced apoptosis. Human NAG-1 cDNA was transfected into KB cells. NAG-1 overexpression caused increased caspase-3 cleavage and cleaved PARP, and treatment of KB/NAG-1 with 40 µM TA further enhanced both cleaved caspase-3 and cleaved PARP, compared to TA-treated control cells (Fig. 4A). In parallel with increased caspase-3, KB/NAG-1 cells markedly increased typical morphological features of apoptosis, including Annexin V-positive and TUNEL-positive cell staining, compared to control cells. This effect was further increased by TA treatment (Fig. 4B and 4C). We then examined whether suppression of NAG-1 expression could modulate TA-induced apoptosis. KB cells transfected with either NAG-1 siRNA or control siRNA were treated with or without 30 µM TA for 24 h. Immunoblot analysis demonstrated that transfection of siRNA against NAG-1 suppressed expression of NAG-1, cleaved PARP and cleaved caspase-3 in the presence of TA, compared to control transfected cells (Fig. 5A). Under these conditions, TA-mediated Annexin V-positive and TUNEL-positive cells were significantly attenuated in cells transfected with NAG-1 siRNA (Figs. 5B and 5C). Taken together, these results suggest that NAG-1 plays a role in apoptosis and that inhibition of NAG-1 in KB cells affect TA-induced apoptosis. Moreover, these results suggest that TA-induced NAG-1 up-regulation may be one of the underlying mechanisms that contribute to TA-induced apoptosis.

### Effect of TA on tumorigenicity and apoptosis in BALB/c nu/nu mice

**Figure 6:**
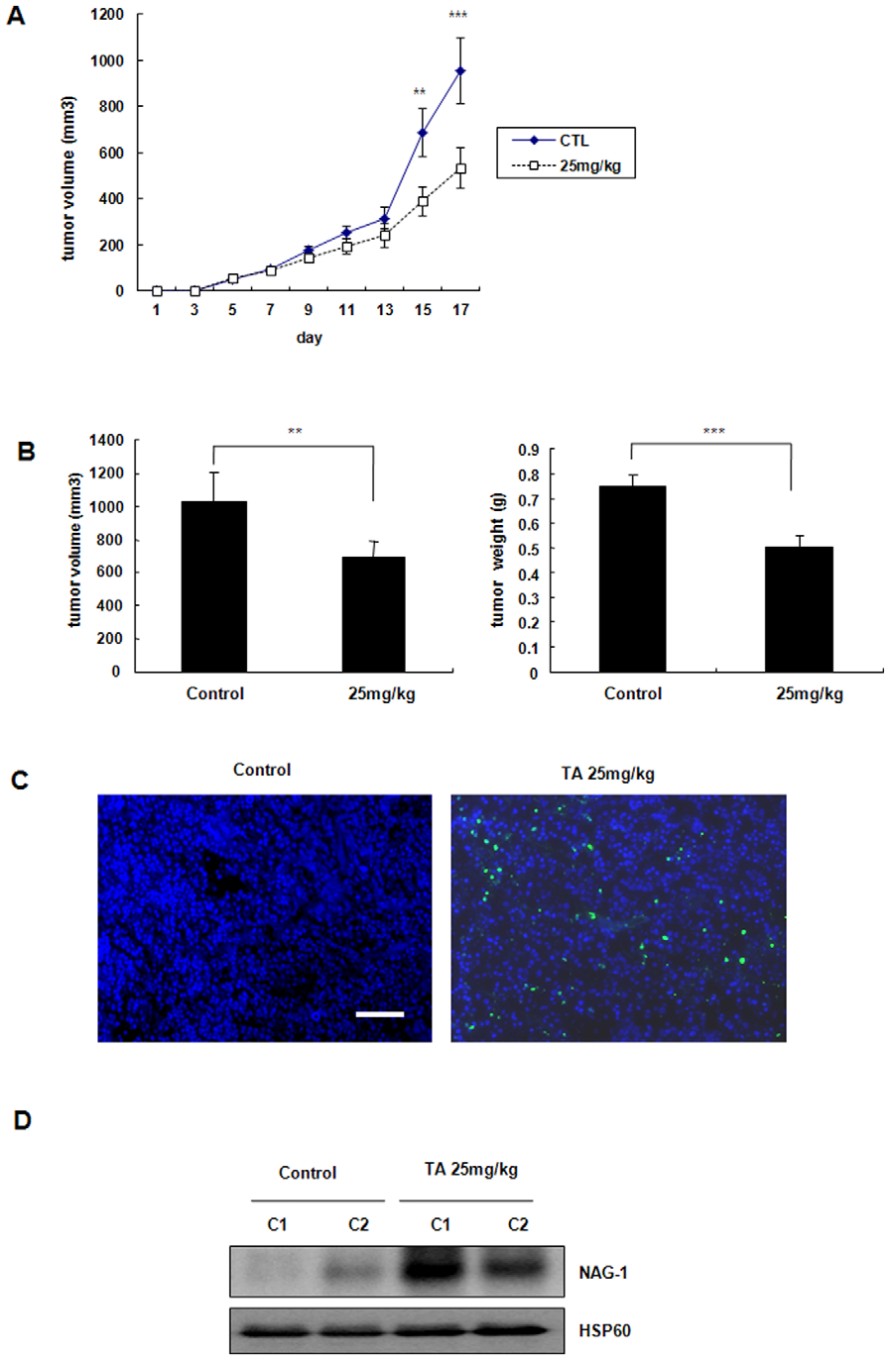
Effect of TA on antitumor effect and *in vivo* expression of NAG-1 in BALB/c nu/nu mice. (A) BALB/c nu/nu mice were randomly divided into two equal groups (control and 25 mg/kg) after injection of KB cells. After tumor formation (around 100 mm in diameter), treatment was started with intraperitoneal injection of either TA (25 mg/kg) daily (treatment groups) or an equal volume of saline alone (control group). Animals were killed and tumors were collected and weighed 17 days after implantation. (B) Graph of the volume and weight of tumors. (C) TUNEL staining in control and treatment groups. The scale bar denotes 50 µm. (D) NAG-1 expression in implanted KB tumors. The induction of NAG-1 by TA is compared to each control.

To determine whether the aforementioned results were evident *in vivo*, we used the BALB/c nu/nu mice and randomly divided them equally into the control group and treatment (25 mg/kg TA) group after tumor formation (approximately 100 mm in diameter). All mice survived throughout the experimental period. Administration of TA significantly affected tumor formation and development (*p*<0.05; Figs. 6A and 6B). However, there was no significant difference between the two groups in body weight. TUNEL staining revealed that TA significantly increased the number of TUNEL positive cells (Fig. 6C). To investigate the level of NAG-1 in the tumors, Western blotting of NAG-1 expression on tumor specimens of BALB/c nu/nu mice was performed. Strong induction of *in vivo* NAG-1 expression in proteins that were extracted from tumors in TA-treated mice was evident, compared to control mice. (Fig. 6D) The data suggest that TA increases the expression of NAG-1 in tumors and that the induced NAG-1 suppresses the size of tumors.

## Discussion

NSAIDs inhibit COX-dependent synthesis of prostaglandins that mediate inflammatory responses in multiple tissues [3], [4], [5]. NSAIDs also induce cell growth inhibition, apoptosis, and antiangiogenesis; activation of the pathways involved in these effects is critical for the reported anticarcinogenic activities of NSAIDs and related COX-2 inhibitors, such as celecoxib [17], [18], [19], [20], [21]. Extensive epidemiologic studies of the role of NSAIDs in the prevention and treatment of colon cancer has provided evidence that the use of NSAIDs, such as aspirin and some COX-2 inhibitors, is associated with a decrease in the incidence and/or mortality of colon cancer [22], [23], [24].

Recently, it was reported that NSAIDs have other targets in addition to COX-2. He et al [25]. reported that the peroxysome proliferation activating receptor delta (PPARδ) is also an adenomatous polyposis coli (APC)-regulated target of NSAIDs. In addition, NSAIDs metabolites can suppress the nuclear factor-κB (NF-κB) survival signal via I κB kinase α (IκKα) inhibition [26]. These suggest that NSAIDs inhibit tumor growth via COX-dependent and -independent pathways.

TA is a potent, well-tolerated NSAID with a low gastric side effect and high therapeutic index, as compared with other NSAIDs [27]. It possesses antipyretic and analgesic activities as evidenced in several animal models and has shown promising results in long-term treatment of osteoarthritis, rheumatoid arthritis, and migraine [27]. TA also affects apoptosis in colorectal cancer cells, as well as metastasis and tumorigenesis in pancreatic cancer models [28] [29], [30].

Specificity protein 1 (Sp1) degradation by TA inhibits several cancer cell lines including pancreatic cancer cells [28], [29]. Thus, Sp1 can be another possible molecular target for TA-induced apoptosis. TA also decreased several Sp-dependent genes and proteins such as vascular endothelial growth factor (VEGF), VEGFR1, survivin, cyclin D1, and bcl-2, and these responses contribute to their anticancer activity [31]. Recently, Kim et al. showed that COX-1 and COX-2 proteins were not changed by TA and that TA induced cell growth inhibition occurred in a COX-independent manner in KB HNSCC cells [32].

Despite these advancements in knowledge, the molecular mechanism of TA on antitumorigenesis in HNSCC is not clear. It is likely that multiple mechanisms are operative. The effects of TA on the growth of KB human cancer cells and the mechanism underlying these effects were presently investigated.

In this study, we demonstrated that NSAIDs induce NAG-1 expression and apoptosis in HNSCC cells, and that TA is the most potent NSAID of those tested. Although the induction of apoptosis by NSAIDs has been observed in several cancer cells [10], [11], [33], [34], [35], the specific NSAID that causes maximal induction of NAG-1 expression depends on the type of cancer cell. For example, sulindac sulfide is the most potent NAG-1 inducer in colorectal cancer and indomethacin is the most potent in sinonasal cancer [5], [10], [36] These differences are probably related to the complex regulation of NAG-1 expression, which involves both transcriptional and posttranscriptional mechanisms. Furthermore, its expression seems to involve cis- and trans-acting promoter elements, various transcription factors, and several antitumorigenic substances [37]. NAG-1 is also a downstream target of the diverse p53, ERG-1 and AKT/GSK-3 tumor suppressing pathways [12], [13], [14], [38], [39], [40], [41]. Thus, the mechanism of NAG-1 regulation depends on cell type and the mode of induction. Further studies on the induction of NAG-1 by TA in HNSCC cells are necessary.

The present data demonstrate that NAG-1 is one of the key regulators for TA-induced apoptosis. TA inhibited the growth of KB cells (Fig. 1). A higher concentration of TA (100 µM) resulted in more than 70% cell death, with cells becoming detached and rounded-up within 24 h after treatment.

In addition, TA induced apoptosis as evidenced by the increased sub-G1 population, nucleosomal fragmentation (data not shown), and cleaved PARP (Fig. 2), suggesting that apoptosis contributes to the inhibitory effects of TA on the viability of KB cells. These results agree with previous reports that TA induces PARP cleavage in colorectal cancer cells [30] and pancreatic cancer cells [28].

Mitochondrial damage manifests as an initial hyperpolarization, followed by a loss of the mitochondrial membrane potential and the release of proteins from the mitochondrial intermembrane space, such as cytochrome c. Direct and indirect perturbation of the mitochondria can activate the apoptotic pathway. TA significantly induced the loss of MMP (Fig. 3), suggesting that mitochondrial damage is involved in TA-induced apoptosis.

NAG-1 was induced by TA in a dose- and time-dependent manner (Fig. 2), and overexpression of NAG-1 enhanced the apoptotic effect of TA (Fig. 4). Moreover, siRNA-mediated knockdown of NAG-1 attenuated TA-induced apoptosis (Fig. 5), suggesting that NAG-1 expression is closely associated with apoptosis.

The *in vivo* anticarcinogenic activity of TA was investigated in athymic nude mice bearing KB cells as xenografts. At a dose of 25 mg/kg/day, TA significantly inhibited tumor growth and weight and increased TUNEL positive staining cells (apoptosis) in tumor sections from treated versus control animals. This was accompanied by increased NAG-1 staining in tumors from treated mice (Fig. 6). Clinical studies with TA for chronic treatment of rheumatoid arthritis has used doses of TA as high as 10 mg/kg/day, which is only 2.5-fold lower than the dose required for inhibition of HNSCC tumor growth in a xenograft mouse model. These results demonstrate that TA is a highly effective anticancer agent in both *in vitro* and *in vivo* models of HNSCC, and complements previous results [29], [31], [32]. The mechanism of action of TA as a tumor growth inhibitor is due, in part, to induction of NAG-1 and NAG-1-related genes.

The linkage between NAG-1 induction and TA revealed in this study provides a new molecular mechanism that may contribute to the anti-tumorigenic activities of TA in HNSCC.
